# Ramsey theory and thermodynamics

**DOI:** 10.1016/j.heliyon.2023.e13561

**Published:** 2023-02-10

**Authors:** Nir Shvalb, Mark Frenkel, Shraga Shoval, Edward Bormashenko

**Affiliations:** a Department of Mechanical Engineering & Mechatronics, Faculty of Engineering, Ariel University, P.O.B.3, Ariel, 407000, Israel; b Chemical Engineering Department, Engineering Faculty, Ariel University, P.O.B. 3, 407000 Ariel, Israel; c Department of Industrial Engineering and Management, Faculty of Engineering, Ariel University, P.O.B. 3, Ariel 407000, Israel

**Keywords:** Thermodynamics, Ramsey theory, Graph theory, Directed graph, Irreversible process

## Abstract

Re-shaping of thermodynamics with the graph theory and Ramsey theory is suggested. Maps built of thermodynamic states are addressed. Thermodynamic states may be attainable and non-attainable by the thermodynamic process in the system of constant mass. We address the following question how large should be a graph describing connections between discrete thermodynamic states to guarantee the appearance of thermodynamic cycles? The Ramsey theory supplies the answer to this question. Direct graphs emerging from the chains of irreversible thermodynamic processes are considered. In any complete directed graph, representing the thermodynamic states of the system the Hamiltonian path is found. Transitive thermodynamic tournaments are addressed. The entire transitive thermodynamic tournament built of irreversible processes does not contain a cycle of length 3, or in other words, the transitive thermodynamic tournament is acyclic and contains no directed thermodynamic cycles.

## 1 Introduction

In 1900 David Hilbert presented a list of twenty-three problems that in his opinion would and should occupy the efforts of mathematicians in the future [1]. The sixth problem of the list deals with the axiomatization of physics [1]. Hilbert suggested “to treat in the same manner (as geometry), by means of axioms, those physical sciences in which mathematics plays an important part” [1]. This problem remains unsolved. The general fundamental physical axiomatic system does not exist. However, one of the branches of physics, namely thermodynamics, enables the self-consistent axiomatic formulation. The first successful attempt to reshape thermodynamic into the axiomatic theory was carried out by Constantin Carathéodory [2]. Carathéodory understood that just thermodynamics is well-suited for axiomatization. In particular, equations of thermodynamics, known as the First Law of Thermodynamics appear in a linear differential form that is known for mathematicians as Pfaff expressions or Pfaff differential forms, enabling a formal mathematical analysis of these equations [2,3]. Carathéodory also developed the axiomatic approach to the Second Law of Thermodynamics formulated as follows: “In the neighborhood of any equilibrium state of a system (of any number of thermodynamic coordinates), there exist states that are inaccessible by reversible adiabatic processes [3,4]”. The aforementioned formulations of thermodynamics highlighted the role of the binary relation of adiabatic accessibility between two thermodynamic states [5]. The idea that the binary relations interconnecting thermodynamic states are crucial for constituting thermodynamics hints to the hypothesis that the Ramsey Theory may be useful for the “mathematical thermodynamics”.

Ramsey theory is a branch of graph theory that focuses on the appearance of interconnected substructures within a structure/graph of a known size [[6], [7], [8], [9], [10], [11], [12], [13]]. Ramsey theory states that any structure will necessarily contain an interconnected substructure [[7], [8], [9], [10]]. From the mathematical point of view Ramsey theorem provides generalization of the pigeonhole principle, stating that if *n* items are put into *m* containers, with n>m, then at least one container must contain more than one item [[7], [8], [9], [10], [11], [12], [13]]. Ramsey's theorem, in one of its graph-theoretic forms, states, in turn, that one will find monochromatic cliques in any edge labelling (with colors) of a sufficiently large complete graph [7]. A clique is defined as a subset of vertices of an undirected graph such that every two distinct vertices in the clique are adjacent [7]. To demonstrate the theorem for two colors (say, green and red), let *n* and *k* be any two positive integers, the Ramsey theorem states that there exists a least positive integer R(n,k) for which every green-red link coloring of the complete graph on R(n,k) vertices contains a green clique on *n* vertices or a red clique on *k* vertices. The Schur Theorem, stating for any r∈N there is a natural number *M* such that any *r*-coloring of [1, *M*] contains *x*, *y*, *z* having the same color such that x+y=z, is an example of the Ramsey-inspired-thinking. One more example of the Ramsey-like thinking is delivered by the van der Waerden's theorem: colorings of the integers by finitely many colors must have long monochromatic arithmetic progressions [7]. An accessible introduction to the Ramsey theory is found in refs. 7, 9. More rigorous approach is laid out in refs. 10–11. Applications of the Ramsey theory for the theory of communication are addressed in ref. 13. Problems in Ramsey theory typically ask a question of the form: "how big must some structure be to guarantee that a particular property holds?" We re-shape this question for thermodynamics in a following form: how large should be a graph describing connections between discrete thermodynamic states to guarantee the appearance of thermodynamic cycles (which are crucial for modern thermodynamics) [14,15]? Ideas, emerging from the graph theory entered recently into chemistry [[16], [17], [18], [19]]. We demonstrate that these ideas may be introduced effectively into thermodynamics. Thermodynamics is usually seen as a theory, which deals with continuous macroscopic variables. Ramsey-theory-inspired thinking is a step in the direction of development of discrete thermodynamics, which was introduced recently [20,21].

## 2 Ramsey theory and unattainable thermodynamic states

Consider the map of thermodynamic states inherent for the thermodynamic system of constant mass, namely, a thermodynamic system enclosed by walls through which mass cannot pass, however heat could be delivered to the system [14]. Thermodynamic states of the system are fully identified by values of a triad of parameters known as state variables, namely: pressure, volume and temperature (P,V,T). The addressed map contains six thermodynamic states and it is depicted in Fig. 1. The map built of six discrete thermodynamic vertices is actual in a view of the recent development of six-stroke-engines, demonstrating better engine performance and emissions than four-stroke-machines [[22], [23], [24]]. Six discrete thermodynamic states are distinguished in the cycle of these engines (see Fig. 2).

**Fig. 1:**
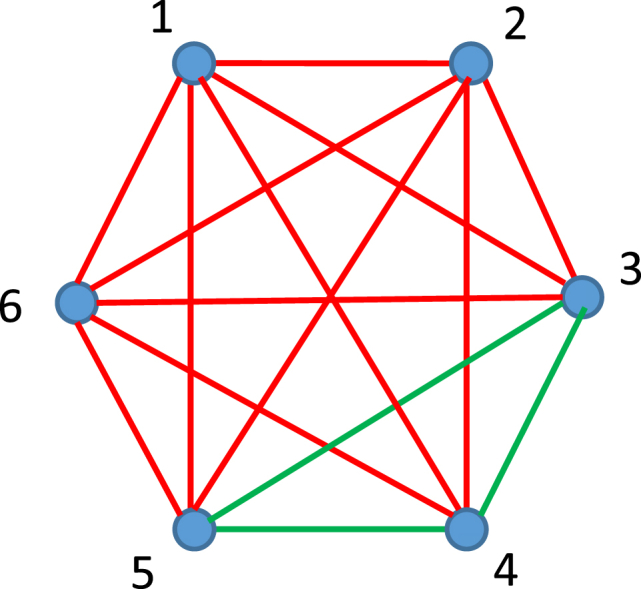
The map of thermodynamic states available for the constant mass thermodynamic system is depicted. Red lines connect the states which are attainable by the thermodynamic process. Green lines connect the states which are unattainable by the thermodynamic process. Cyclic process “124”, “146”, “256” and “345” are recognized in the map.

**Fig. 2:**
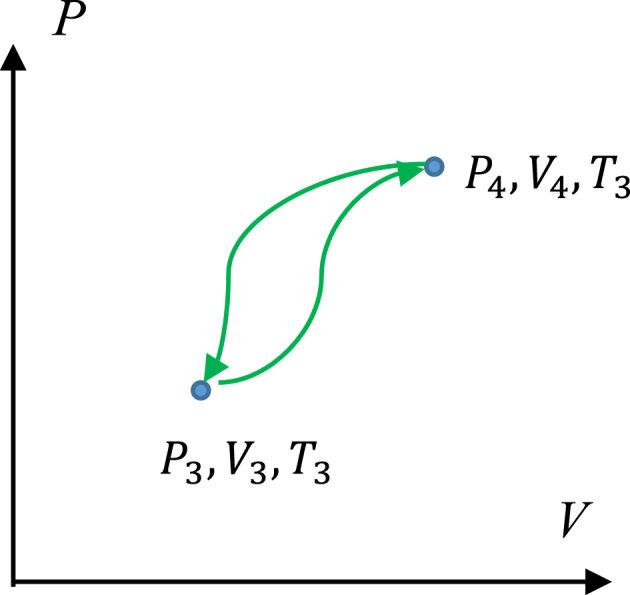
The processes impossible in the thermodynamic system of constant mass are depicted. ${\left(\frac{\partial P}{\partial V}\right)}_{T}\geq 0$ is true for the pathways 3→4 and 4→3.

Vertices depict certain discrete thermodynamic states $\left(P_{i},V_{i},T_{i},i=1\ldots 6\right)\text{.}$ Two kinds of interrelations between the points numbered correspondingly *n* and *k* are possible, namely: i) the process n→k corresponding to the transition $\left(P_{n},V_{n},T_{n})\to (P_{k},V_{k},T_{k}\right)$ is thermodynamically available (these points are connected with red lines), and ii) the process n→k corresponding to the transition $\left(P_{n},V_{n},T_{n})\to (P_{k},V_{k},T_{k}\right)$ is thermodynamically unattainable (these points are connected with green lines in Fig. 1).

Why a thermodynamic transition (process) may be unavailable? The thermodynamic process in the system of the constant mass is possible, when Eq. (1) is fulfilled:

(1) $${\left(\frac{\partial P}{\partial V}\right)}_{T}<0$$

Eq. (1) means that the pressure and volume in the closed thermodynamic system cannot grow simultaneously within the isothermal process, and it is equivalent to the condition $C_{V}>0$, where $C_{V}$ is thermal capacity of the system under constant volume [14].

Eq. (1) actually emerges from the Second Law of Thermodynamics, and it is true for homogeneous physical systems [14]. For example, the transition from point “3” to point “4” (see Fig. 1) corresponds to the isothermal process, for which Eq. (2) is true:

(2) $$P_{4}>P_{3};V_{4}>V_{3};T=const$$

The algorithmic procedure of ordering of thermodynamic states is supplied in Appendix A. Processes (pathways) which are impossible in the closed thermodynamic systems are illustrated with Fig. 2.

The isothermal processes 3 → 4 and 4 → 3 are impossible in the thermodynamic system m=const), due to the fact that ${\left(\frac{\partial P}{\partial V}\right)}_{T}\geq 0$ takes place along the aforementioned pathways, shown in Fig. 2. And it should be emphasized that both the “direct” 3 → 4 and “reverse” 4 → 3 processes are thermodynamically unattainable in the constant mass system [14].

Thus, two kinds of relationship are possible within the thermodynamic map depicted in Fig. 1. These relationships form the complete graph, i.e. a graph in which each pair of thermodynamic states are connected by an edge, depicting the possibility of thermodynamic transition between the states. It is noteworthy, that any thermodynamic map, such as depicted in Fig. 1, forms a complete graph, indeed, from a pure logical point of view, the thermodynamic transition from state labeled “*n*” to the state labeled “*k*” is or thermodynamically possible or alternatively, impossible. Thus, the ideal conditions for the application of the Ramsey theory to the analysis of the aforementioned thermodynamic maps/graphs are created.

We recognize a number of triangles, namely, the red triangles: “164”, “162”, “125”. “136” and the green one “345” in Fig. 1. Thus, a number of cyclic processes appear at our thermodynamic map. The green triangle “345” corresponds to the thermodynamically impossible cycle, whereas the cyclic process “164” is available for the system. The importance of cyclic processes for thermodynamics is crucial [14,15]. Let us ask the following fundamental question: what is the minimal number of points at our thermodynamic map in which three-step (e.g. three-stroke) cyclic processes (possible or forbidden) will necessarily appear? Why three-stroke thermodynamic processes are important? It was demonstrated recently that the optimal efficiency and work production per cycle within the whole class of irreversible minimal-coupling engines may be achieved for the-three stroke quantum engine and with the two-quantum-level working body [25]; the efficiency of such an engine will be addressed below. The minimal reversible classical cyclic process, enabling extraction of mechanical work also includes three points at the thermodynamic map. Thus, the question is formulated as follows: what is the minimal number of points on the thermodynamic map guaranteeing the three-stroke cycle should be addressed.

The answer to this question is supplied by the Ramsey theory, and it is formulated as follows: what is the minimal number R(3,3)? The answer emerging from the Ramsey Theory is: R(3,3)=6 (see refs. 7–11). Indeed, we recognize in the example illustrated with Fig. 1, that in the thermodynamic map comprising six points corresponding to distinguishable thermodynamic states, in which the relationships “to be thermodynamically attainable” and “to be thermodynamically unattainable” are necessarily present we find triads of states forming the cyclic processes, some of which corresponds to the attainable and the other to the unattainable cycles (consider that possible *or* impossible cycles will necessary appear at the map). If the thermodynamically the attainable cycle “164” is present in the map, its maximal efficiency $\eta_{\max}$ is given by Eq. (3):

(3) $$\eta_{\max}=1-\frac{T_{\min}}{T_{\max}}$$

where $T_{\min}=\min\left\{T_{1},T_{4},T_{6}\right\}$ and $T_{\max}=\max\left\{T_{1},T_{4},T_{6}\right\}$ correspondingly. The maximal efficiency of the three-stroke quantum engine exploiting the two-quantum-level working body, was derived in ref. 25, as supplied in Eq. (4):

(4) $$\eta_{\max}=1-\frac{e^{\frac{\omega }{k_{B}T_{\max}}}-1}{1-e^{-\frac{\omega }{k_{B}T_{\min}}}}$$

where $k_{B}$ is the Boltzmann constant and ω is the energy gap of the two-level system. In the limit $\frac{\omega }{k_{B}T}\to 0$ the Carnot efficiency, given by Eq. (3) is achieved [25]. It should be emphasized, that the thermodynamically possible cycle are not necessarily present in the thermodynamic map. Simple, algorithmic procedure enabling ordering of the thermodynamic states is supplied in Appendix A.

Let us take a more close thermodynamic look on the thermodynamically attainable processes such as process 1→2; the reverse process 2→1 is also latently suggested to be available; thus, process 1→2 is reversible; from the logical point of view it means that thermodynamic attainability is the commutative property for the thermodynamic systems of a constant mass.

## 3 Theory of graphs and irreversible thermodynamic processes

**Fig. 3:**
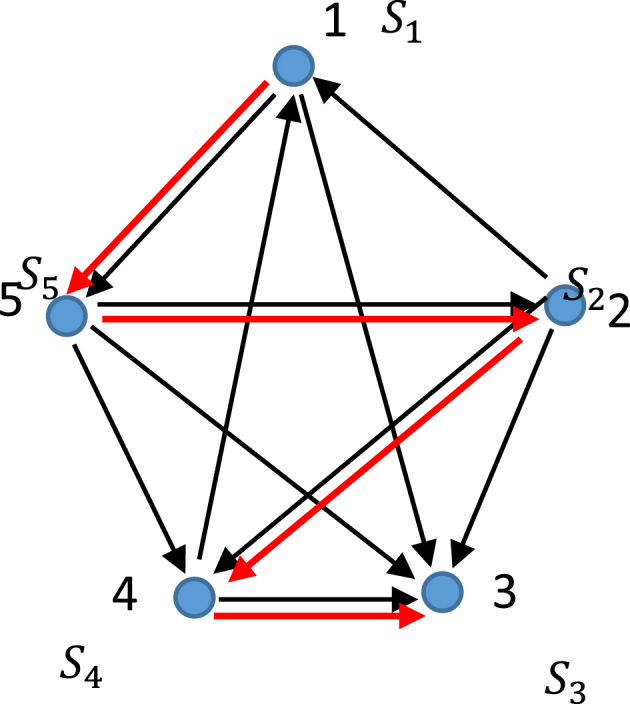
Vertices on the graph represent thermodynamic states $\left(P_{i},V_{i},T_{i},i=1\ldots 5\right)$. Only irreversible transitions between the states depicted by black arrows are possible. Red arrows demonstrate the Hamiltonian path.

Now consider the thermodynamic map shown in Fig. 3. Again, every point depicts a certain thermodynamic state $\left(P_{i},V_{i},T_{i},i=1\ldots 5\right)\text{.}$ Now, only irreversible processes occurring between the states are possible. We define the processes as “irreversible”, when they create new entropy [15].

The thermodynamic states, shown in Fig. 3 form a "tournament" which is a directed graph obtained by assigning a direction for each edge in an undirected complete graph [[26], [27], [28]]. Any tournament on a finite number *n* of vertices contains a Hamiltonian path, i.e., directed path on all *n* vertices (Hamiltonian or traceable path is a path in an undirected or directed graph that visits each vertex exactly once [[26], [27], [28]]), which is shown with red arrows in Fig. 3. From the physical point of view, the pathway 15,243 illustrates irreversible process involving all of the possible thermodynamic states. It should be emphasized, that in any complete directed graph, representing the thermodynamic states of the system the Hamiltonian path is found. It should be mentioned, that the entropy is a relativistic invariant [29]; thus, the Hamiltonian path, shown in Fig. 5, is the relativistic invariant of the thermodynamic graph in which entropies represent the vertices.

The tournament may be transitive, in other words: ((a→b)and(b→c))⇒(a→c) takes place in such a tournament [30]. From the physical point of view this means, that the irreversible transition 1→2 followed by the irreversibly transition 2 →3 implies the availability of the irreversible transition 1 →3. If the thermodynamic states, form the map, in which only irreversible transitive processes are possible, they form the transitive tournament. This necessarily means that the entire tournament contain a no directed thermodynamic cycles. Consider, that real thermodynamic processes are always irreversible to some extent; thus, we proved that no directed thermodynamic cycles are possible in real thermodynamic systems, in which entropy growth is inevitable. This statement actually presents the re-formulation of the Second Law of Thermodynamics, as worded with the graphs theory notions.

**Fig. 4:**
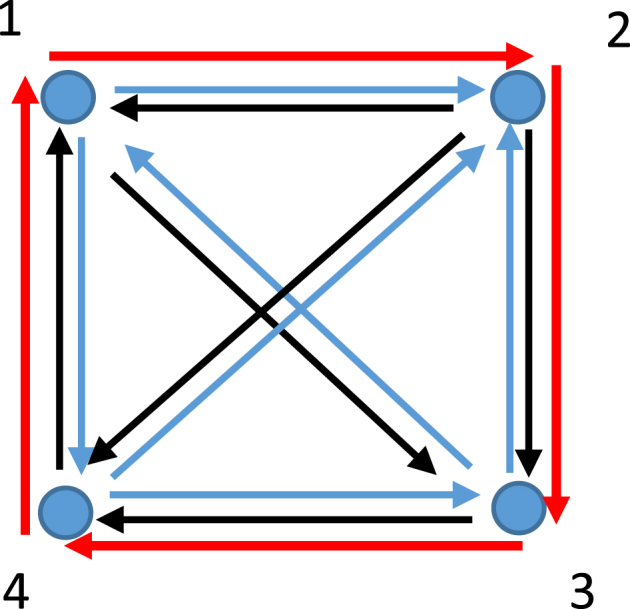
The strongly connected graph connected four thermodynamic states is shown. Red path depict the Hamiltonian cycle inherent for this thermodynamic map.

Thermodynamic states may also form the "strongly connected graph", namely a graph in which every vertex is reachable from every other. Such a graph for a map of thermodynamic states $\left(P_{i},V_{i},T_{i},i=1\ldots 4\right)$ is shown in Fig. 4.

**Fig. 5:**
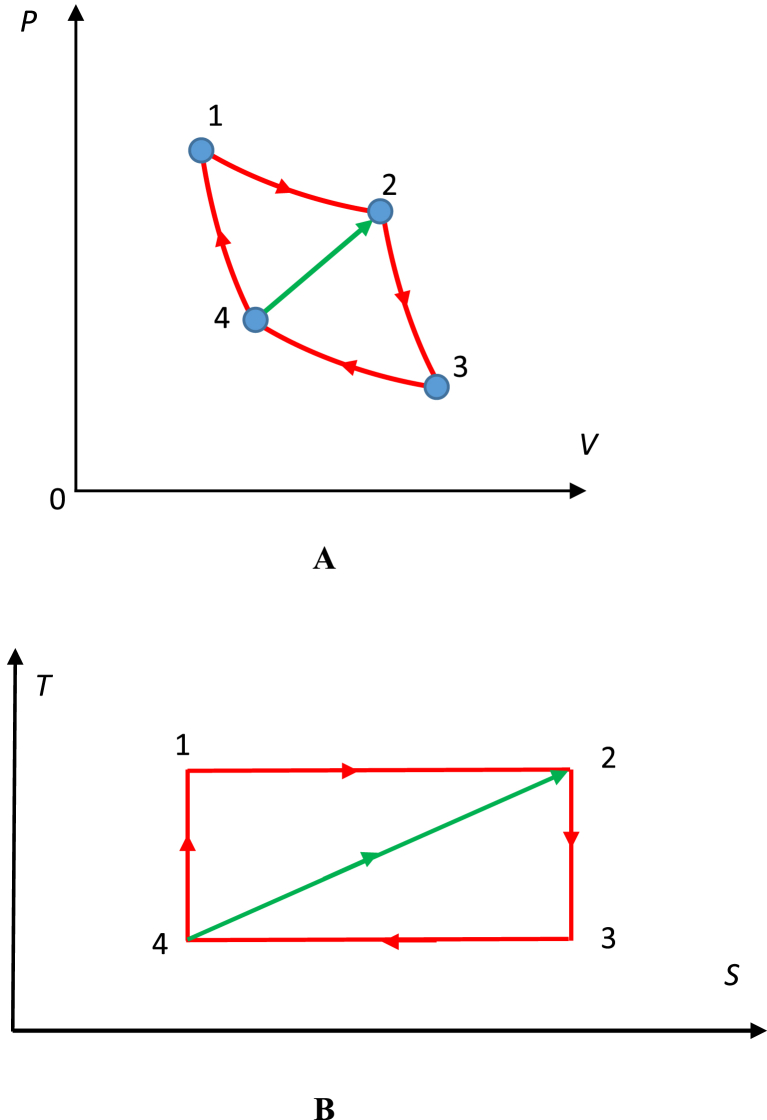
**A**. *PV* representation of the Carnot cycle. **B**. *TS* representation of the Carnot cycle. The Carnot cycle 12,341 may be seen as the strongly connected graph; red solid line is the cyclic Hamiltonian path; the green arrow depicts the “forbidden thermodynamic process”.

Every strongly connected tournament has a Hamiltonian cycle, which is shown with the red path in Fig. 4. The opposite is also true: if the graph has a Hamiltonian cycle it is strongly connected. Cyclic reversible thermodynamic processes (such as the Carnot cycle) are seen in this context as strongly connected graphs, as shown in Fig. 5A and B, depicting *PV* and TS diagrams of the cycle. It is noteworthy that this graph *1234* is not complete and the edge *42* represents the process, which is forbidden within the constant mass system.

Now let us return to the Caratheodory axiomatic thermodynamics. Consider first the thermodynamic map representing the states, which all are attainable by reversible adiabatic processes within the system of the constant mass.

**Fig. 6:**
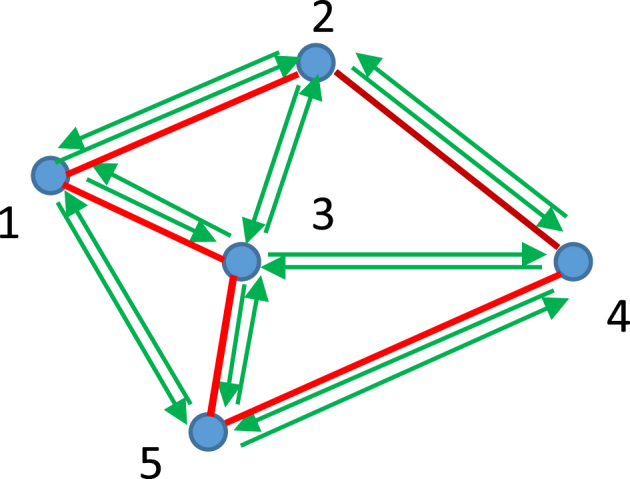
The graph presents five thermodynamic states between which reversible adiabatic processes are possible. Path *124,351* depicts the Hamiltonian cycle for this map.

This graph is strongly connected and necessarily contains the Hamiltonian cycle *124,351* shown in Fig. 6.

Now consider the “mixed” map of states, which contains both kinds of thermodynamic processes, namely attainable and non-attainable with the reversible adiabatic process. Two types are possible for such graphs: i) non-transitive tournaments; ii) transitive tournaments. When three thermodynamic states are located on the same adiabatic curve, we deal with the transitive tournament. The transitive tournament contains no directed cycles and this is true for any number of vertices/thermodynamic states forming the complete graph [30]. Non-transitive complete graphs were discussed in Section 2. Applications of the Ramsey theory to physical problems are sparse, yet [31,32]. A classical Hamiltonian that favors configurations in a way to establish lower bounds on Ramsey numbers was reported in ref. 31. Ramsey Theory supplies the selection rules for the vibrational spectra of the cyclic molecules [32]. Application of the Ramsey theory to the systems, in which the attraction and repulsion forces act, is reported in ref. [33]. We demonstrate the possibility of applications of the Ramsey approach in thermodynamics.

## 4 Conclusions

Equilibrium thermodynamics is a branch of classical physics, which enables rigorous axiomatic mathematical treatment, such as axiomatic thermodynamics, suggested by Carathéodory. We demonstrate that the equilibrium thermodynamic may be re-shaped with the graph theory, in particular, exploiting the approaches developed within the Ramsey theory. The application of the Ramsey theory becomes possible when maps built of thermodynamic states are addressed. Ramsey theory enables treatment of the following problem: how large should be a graph describing connections between discrete thermodynamic states to guarantee the appearance of thermodynamic cycles, which play a crucial role in the classical thermodynamics. We introduce the following approach: thermodynamic states may be attainable and non-attainable by the thermodynamic process in the system of constant mass. The thermodynamic process in the system of the constant mass is possible, when the condition ${\left(\frac{\partial P}{\partial V}\right)}_{T}<0$ is fulfilled. Thus, the processes for which ${\left(\frac{\partial P}{\partial V}\right)}_{T}>0$ are forbidden; this makes possible the construction of the complete graph, connecting the states constituting the thermodynamic map. The Ramsey theory states that in the thermodynamic map comprising six or more points corresponding to distinguishable thermodynamic states, in which the relationships “to be thermodynamically attainable” and “to be thermodynamically unattainable” are present, we necessarily find at least one triad of states forming the cyclic processes, one of which corresponds to the attainable and the other to the unattainable cycles. This statement is useful for the analysis of six-stroke thermal engines, introduced recently. Direct graphs emerging from the sequences of irreversible thermodynamic processes are considered. In any complete directed graph, representing the thermodynamic states of the system the Hamiltonian path, i.e. directed path on all *n* vertices/states is found. The Hamiltonian path of the tournament emerging from the graph uniting the vertices, representing the entropy of discrete thermodynamic states, is a relativistic invariant. Transitive thermodynamic tournaments are addressed. The entire transitive thermodynamic tournament built of irreversible processes does not contain a cycle of length 3, or in other words the transitive thermodynamic tournament is acyclic and contains no directed thermodynamic cycles. This statement supplies the alternative shaping of the Second Law of Thermodynamics. Shortcomings, inherent to the Ramsey theory, should be considered. Firstly, the results supplied by the Ramsey theory are non-constructive: they may show that some sub-structure exists, but they give no process for finding this structure (other than brute-force search). Secondly, the Ramsey theory states that sufficiently large graphs must necessarily contain a given sub-structure, often the proof of these results requires these objects to be enormously large, giving rise to bounds that grow exponentially. Anyway, the Ramsey approach enables a fresh glance on the thermodynamic processes seen as graphs and reshaping of thermodynamic problems with the notions of mathematical logic.

## Authorship contribution statement

Dr Nir Shvalb; Dr Mark Frenkel: Conceived and designed the analysis; Analyzed and interpreted the data; Contributed analysis tools or data.

Dr Shraga Shoval; Dr Edward Bormashenko: Conceived and designed the analysis; Analyzed and interpreted the data; Contributed analysis tools or data; Wrote the paper.

## Funding statement

This research did not receive any specific grant from funding agencies in the public, commercial, or not-for-profit sectors.

## Data availability statement

Data included in article/supplementary material/referenced in article.

## Declaration of interest’s statement

The authors declare no conflict of interest.

## Figures and Tables

**Table 1A:** Thermodynamic states available to the thermodynamic system of the constant mass.

	P_1_	P_2_	P_3_
*V*_*1*_	*P*_*1*_*V*_*1*_	*P*_*2*_*V*_*1*_	*P*_*3*_*V*_*1*_
*V*_*2*_	*P*_*1*_*V*_*2*_	*P*_*2*_*V*_*2*_	*P*_*3*_*V*_*2*_
*V*_*3*_	*P*_*1*_*V*_*3*_	*P*_*2*_*V*_*3*_	*P*_*3*_*V*_*3*_

Some of transitions between these states will be thermodynamically attainable a some of them are non-attainable. The complete Ramsey graph depicted the states is shown in Fig. 1A. Red solid lines depict possible transitions and blue ones demonstrate “forbidden” ones.

**Fig. 1A:**
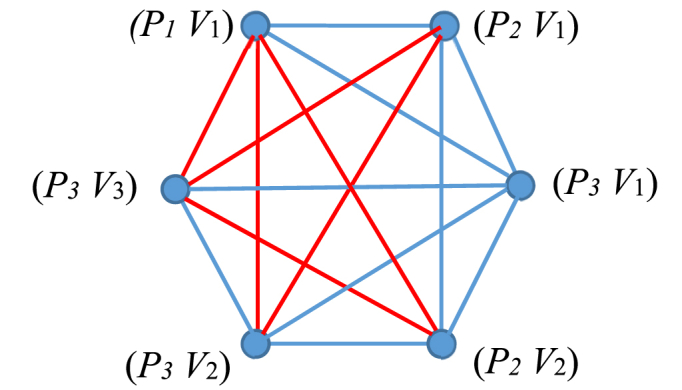
The map of thermodynamic states available for the constant mass thermodynamic system is depicted; $P_{1}>P_{2}>P_{3};\;V_{1}<V_{2}<V_{3}$. Red lines connect the states which are attainable by the thermodynamic process. Blue lines connect the states which are unattainable by the thermodynamic process.

**Fig. 2A:**
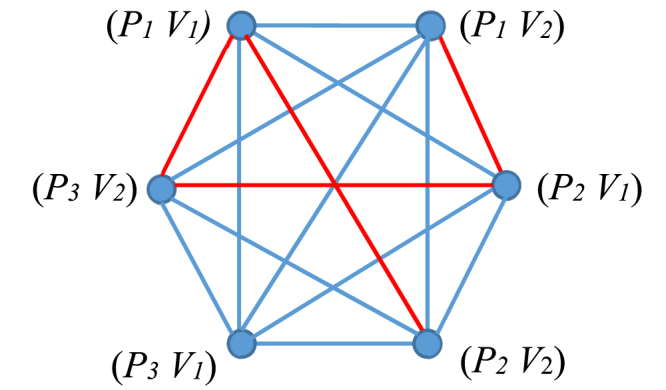
The map of thermodynamic states available for the constant mass thermodynamic system is depicted; $P_{1}>P_{2}>P_{3};\;V_{1}<V_{2}<V_{3}$. Red lines connect the states which are attainable by the thermodynamic process. Blue lines connect the states which are unattainable by the thermodynamic process.

## Appendix A Procedure enabling ordering of the thermodynamic states suitable for the Ramsey analysis

The states available for the thermodynamic system of the constant are supplied in Table 1A. The pressure and volume of the states are ordered as follows: $P_{1}>P_{2}>P_{3};\;V_{1}<V_{2}<V_{3}$. The thermodynamic states are summarized in Table 1A.

Available $\left(P_{1}{,V}_{1}\right)\to \left(P_{2},V_{2}\right)\to \left(P_{3},V_{3}\right)$ and forbidden $\left(P_{1}{,V}_{1}\right)\to \left(P_{2},V_{1}\right)\to \left(P_{3},V_{1}\right);\;\left(P_{3}{,V}_{1}\right)\to \left(P_{2},V_{2}\right)\to \left(P_{3},V_{2}\right);\;\left(P_{3}{,V}_{3}\right)\to \left(P_{3},V_{1}\right)\to \left(P_{3},V_{2}\right)$ and $\left(P_{2}{,V}_{1}\right)\to \left(P_{3},V_{1}\right)\to \left(P_{2},V_{2}\right)$ cycles are recognized. The available cycle containing five points is also recognized Fig. 2A.

Now we construct the complete graph built of the states: $\left(P_{1},V_{1}\right)\text{,}$ $\left(P_{1},V_{2}\right)\text{,}$ $\left(P_{2},V_{1}\right)\text{,}$ $\left(P_{1},V_{2}\right)\text{,}$ $\left(P_{3},V_{2}\right)\text{,}$ $\left(P_{3},V_{1}\right)$ and $\left(P_{2},V_{2}\right)\text{,}$

In this case, we have no available cycles; however, we have 7 forbidden cycles, for example $\left(P_{1}{,V}_{1}\right)\to \left(P_{2},V_{1}\right)\to \left(P_{3},V_{1}\right);\;\left(P_{1}{,V}_{1}\right)\to \left(P_{1},V_{2}\right)\to \left(P_{3},V_{1}\right)$ and $\left(P_{2}{,V}_{1}\right)\to \left(P_{3},V_{1}\right)\to \left(P_{2},V_{2}\right)$.

According to pressure and volume of the states order and Eq. (1) the cycles are thermodynamically available, when the indices related to pressure and volume decrease and increase simultaneously.
